# Antimicrobial Stewardship During Lactation: Current Evidence on the Human Milk–Infant Microbiome Axis and Neurodevelopment

**DOI:** 10.3390/antibiotics15100970

**Published:** 2026-10-01

**Authors:** Francesco Ferrara, Flavia De Berardinis

**Affiliations:** 1 Pharmaceutical Department, ASL Napoli 3 Sud, Dell’Amicizia Street, 80035 Naples, Italy; 2 Department of Mental Health, ASL Roma 3, Lungomare Paolo Toscanelli, 00121 Rome, Italy; flavia.deberardinis@aslroma3.it

**Keywords:** antimicrobial stewardship, breastfeeding, human milk, lactation, microbiome, infant gut microbiota, neurodevelopment, anti-infective therapy

## Abstract

Human milk is increasingly recognized as a complex biological system that contributes to infant development through nutritional, immunological, and microbial pathways. Simultaneously, antimicrobial stewardship (AMS) has become a global healthcare priority because of rising antimicrobial resistance and concerns regarding the ecological consequences of antibiotic exposure during early life. This narrative review examines current evidence regarding the impact of anti-infective therapies during lactation on the maternal–infant microbiome axis and discusses the role of AMS in supporting appropriate maternal treatment while preserving breastfeeding. While previous reviews have independently described the biological properties of human milk or general antimicrobial resistance, this narrative review uniquely bridges these domains by framing the infant microbiome as a clinical endpoint for pharmacological governance. The primary objective is to synthesize current evidence on the relationships among human milk, the infant gut microbiome, and neurodevelopment, with a novel emphasis on how integrating Antimicrobial Stewardship (AMS) and microbiome-conscious prescribing during lactation actively safeguards these developmental trajectories. This work contributes to the existing literature by shifting the focus from observational biology to an actionable clinical framework for healthcare providers. A structured literature search was conducted in PubMed/MEDLINE, Scopus, and Web of Science. Studies addressing human milk biology, infant gut microbiota development, antimicrobial exposure during lactation, microbiota–gut–brain interactions, and stewardship strategies were reviewed. Available evidence indicates that breastfeeding promotes the establishment of a beneficial gut microbiota and supports immune maturation through the combined action of human milk oligosaccharides, immunological factors, and milk-associated microorganisms. Maternal and neonatal antibiotic exposure may alter microbial colonization patterns; however, the magnitude, persistence, and long-term clinical significance of these effects remain incompletely understood. Although mechanistic studies support biologically plausible links between microbiome alterations and neurodevelopmental pathways, direct evidence connecting maternal antibiotic use during lactation with adverse neurodevelopmental outcomes is limited. Current evidence supports continued breastfeeding during most maternal antibiotic treatments and highlights the importance of microbiome-conscious prescribing. AMS strategies based on diagnostic appropriateness, treatment optimization, and avoidance of unnecessary antibiotic exposure may help minimize microbial disruption while ensuring effective infection management.

## 1. Introduction

The first 1000 days of life, spanning from conception to approximately two years of age, represent a critical period for growth, organ development, immune maturation, and long-term health programming [1,2]. During this developmental window, early nutritional and environmental exposures exert profound effects on physiological systems that may influence health trajectories throughout life. Among these exposures, breastfeeding is widely recognized as one of the most important determinants of infant health and development [1]. Human milk is no longer viewed solely as a source of energy and essential nutrients. It is increasingly recognized as a biologically active and dynamic system containing numerous bioactive components, including human milk oligosaccharides (HMOs), immunoglobulins, antimicrobial proteins, growth factors, extracellular vesicles, non-coding RNAs, and a diverse microbial community [2,3,4,5,6]. These constituents interact with the infant gastrointestinal tract and immune system, contributing to intestinal colonization, epithelial maturation, immune regulation, and host–microbe communication [7,8]. Growing evidence suggests that breastfeeding plays a central role in shaping the infant gut microbiome. Human milk promotes the expansion of beneficial bacterial taxa, particularly Bifidobacterium species, through the combined action of HMOs and milk-associated microorganisms [9,10,11]. This process contributes to microbial ecosystem stability and supports the development of mucosal immune homeostasis during early life [7,11]. In parallel, increasing scientific attention has focused on the microbiota–gut–brain axis, a bidirectional communication network linking intestinal microorganisms with immune, endocrine, metabolic, and neurological pathways [12,13]. Experimental and clinical studies have suggested that microbiome development during infancy may influence neurodevelopmental processes. Furthermore, several observational studies have reported associations between breastfeeding and improved cognitive outcomes, although interpretation of these findings remains complicated by residual confounding factors such as maternal education, socioeconomic status, and family environment [14,15,16,17,18,19]. At the same time, antimicrobial resistance has become a major global public health concern. Inappropriate and excessive antibiotic use contributes not only to the emergence of resistant microorganisms but also to ecological disturbances affecting the human microbiome [20,21]. These concerns are particularly relevant during pregnancy, the neonatal period, and lactation, when microbial colonization patterns are actively developing [22,23]. Antibiotics are among the most frequently prescribed medications during the postpartum period. Although most commonly used agents are considered compatible with breastfeeding from a toxicological perspective, growing interest has emerged regarding their ecological impact on human milk microbiota and infant gut microbial assembly [23,24,25]. Against this background, antimicrobial stewardship has evolved beyond the traditional objective of reducing antimicrobial resistance. In the context of lactation, AMS also aims to optimize therapeutic decisions, minimize unnecessary antimicrobial exposure, preserve breastfeeding whenever possible, and reduce unintended effects on the maternal–infant microbial ecosystem [26,27,28,29]. The primary objective of this narrative review is to evaluate the specific ecological impact of maternal antibiotic use during lactation on the infant gut microbiome, clearly differentiating this indirect exposure from direct neonatal administration or prenatal exposures. The secondary objectives are to critically assess the theoretical and mechanistic implications for the microbiota–gut–brain axis and to define the role of Antimicrobial Stewardship (AMS). By distinguishing established AMS practices from emerging microbiome-oriented hypotheses, this review aims to provide a focused framework for balancing maternal infection management with the preservation of breastfeeding. While numerous published reviews have extensively described the biological benefits of human milk or the broad public health implications of antimicrobial resistance, there remains a critical gap in translating these distinct fields into integrated clinical practice during the postpartum period. Most existing literature treats lactation pharmacotherapy primarily through the lens of acute toxicological safety, often overlooking the ecological collateral damage inflicted upon the infant gut ecosystem. The novelty of this review lies in its translational approach: it bridges the biological complexity of the microbiota–gut–brain axis with the operational principles of Antimicrobial Stewardship (AMS) and clinical governance. Rather than merely presenting previously reviewed findings on human milk components, this work contributes to the existing literature by proposing a “microbiome-conscious prescribing” framework. It repositions the protection of the maternal–infant microbiome not just as a nutritional concern, but as a direct target of rational anti-infective management. In alignment with the focus of this Special Issue, the primary objective of this narrative review is to synthesize current evidence on the role of human milk in shaping infant gut microbiome assembly and neurodevelopment, demonstrating how the optimization of anti-infective therapies during lactation serves as both a One Health infection-control mandate and a crucial neurodevelopmental safeguard. The central contribution is to provide clinicians, pediatricians, and pharmacists with a structured rationale to integrate microbiome preservation into daily therapeutic decision-making within the first 1000 days of life.

## 2. Methods

This structured narrative review was conducted to synthesize evidence on human milk biology, maternal antimicrobial exposure during lactation, and infant microbiome development. A comprehensive literature search was performed in PubMed/MEDLINE, Scopus, and Web of Science, with the final search updated on 15 August 2026. The search strategy utilized the following Boolean string and MeSH terms: (“human milk” OR “breastfeeding” OR “lactation”) AND (“microbiome” OR “microbiota” OR “human milk oligosaccharides”) AND (“anti-bacterial agents” OR “antibiotics” OR “antimicrobial stewardship”) AND (“neurodevelopment” OR “microbiota-gut-brain axis”). Language was restricted to English. Eligibility criteria included systematic reviews, meta-analyses, randomized controlled trials, prospective cohorts, and pharmacokinetic studies published primarily between 2010 and 2026. Studies exclusively analyzing direct neonatal intravenous antibiotics or prenatal (in utero) exposures were excluded to maintain focus on lactational transfer. Two reviewers independently screened titles and abstracts, resolving disagreements through consensus. Following the screening of approximately 450 records, 65 articles were included for full-text data extraction. While a formal systematic Risk of Bias assessment was not performed across all studies, evidence quality was critically appraised based on sample size, prospective design, and the use of multivariable adjustments. For the formulation of clinical recommendations (Table 1), a simplified grading criterion was applied: High (supported by clinical guidelines and meta-analyses), Moderate (supported by consistent observational cohorts), and Low (supported by preliminary, mechanistic, or indirect evidence).

### 2.1. Human Milk as a Biologically Active System

Human milk is universally recognized as the biological reference standard for infant nutrition because it provides not only essential nutrients required for growth but also a highly complex array of bioactive components that support immune maturation, intestinal development, and physiological adaptation during early life. Advances in nutritional sciences, microbiology, and systems biology have progressively transformed the understanding of human milk from a simple source of nourishment into a dynamic biological system capable of mediating communication between the mother and infant [30]. One of the most distinctive characteristics of human milk is its remarkable compositional variability. The concentration of nutrients and bioactive compounds changes throughout lactation, from colostrum to mature milk, and may vary according to maternal genetics, gestational age, health status, environmental exposures, and infant requirements. This variability suggests that human milk functions as a highly adaptive biological fluid that responds to developmental needs during infancy. Consequently, the health benefits associated with breastfeeding are increasingly attributed to the integrated interaction of multiple milk components rather than to individual nutrients acting in isolation [31,32,33,34]. Among these bioactive constituents, human milk oligosaccharides (HMOs) are considered particularly relevant. HMOs constitute the third most abundant solid component of human milk after lactose and lipids and are largely resistant to digestion by the infant gastrointestinal tract. Rather than providing direct nutritional value, they selectively stimulate the growth of beneficial microorganisms, particularly Bifidobacterium species, thereby contributing to the establishment of a characteristic microbial profile observed in breastfed infants [33,34]. Through their prebiotic activity, HMOs support intestinal barrier maturation, microbial community stability, and immune system development during a critical period of host–microbe interaction. In addition to their microbiome-related effects, HMOs may exert direct biological functions on the host. Experimental evidence suggests that they can modulate epithelial cell responses, influence immune signaling pathways, and inhibit pathogen adhesion by acting as soluble receptor analogues [35]. Their structural diversity, including neutral, fucosylated, and sialylated forms, contributes to a wide spectrum of potential biological activities. Nevertheless, although considerable progress has been made in characterizing individual HMOs, the specific contribution of different oligosaccharide profiles to clinically relevant outcomes remains incompletely understood. Human milk also contains a broad spectrum of immunologically active compounds, including secretory immunoglobulin A (sIgA), lactoferrin, lysozyme, cytokines, chemokines, antimicrobial peptides, and growth factors. These molecules contribute to mucosal protection during a period characterized by physiological immune immaturity and increased susceptibility to infection. Secretory IgA plays a key role in immune exclusion at mucosal surfaces, whereas lactoferrin exerts antimicrobial, anti-inflammatory, and immunomodulatory actions that support intestinal homeostasis [5,36]. Collectively, these bioactive factors participate in the development of immune tolerance and the maturation of host defense mechanisms. Another area of expanding scientific interest concerns the microbial component of human milk. Culture-based and culture-independent techniques have demonstrated that human milk contains microbial communities composed of bacteria belonging predominantly to the genera Streptococcus, Staphylococcus, Bifidobacterium, and Lactobacillus [8,9,10]. Although the origin of these microorganisms remains incompletely elucidated, proposed mechanisms include transfer from maternal skin, the infant oral cavity, and potential entero-mammary pathways [7,8]. The concept of a human milk microbiota has generated substantial interest because of its potential role in infant gut colonization. Breastfeeding may contribute to microbial development through multiple complementary mechanisms, including the delivery of beneficial microorganisms, the provision of HMOs that selectively stimulate bacterial growth, and the transfer of immunological factors that shape microbial–host interactions [7,8,9,10,11]. However, despite increasing evidence supporting microbial transfer between mother and infant, the quantitative contribution of milk-derived bacteria to long-term gut microbiome establishment remains a matter of ongoing investigation [10]. Recent studies have also highlighted the presence of extracellular vesicles, microRNAs, long non-coding RNAs, hormones, and bioactive lipids within human milk [37]. These molecules are increasingly viewed as mediators of biological signaling that may influence immune regulation, metabolic programming, and developmental processes [14,15,37]. In particular, extracellular vesicles may facilitate intercellular communication through the transfer of regulatory molecules capable of modulating gene expression in recipient cells. Nevertheless, much of the available evidence remains experimental, and the clinical relevance of these signaling pathways requires further clarification. From a systems-biology perspective, the biological activity of human milk arises from the interaction of nutrients, microorganisms, immunological mediators, and regulatory molecules operating within an integrated ecological network [3,4,30]. This conceptual framework differs substantially from traditional reductionist approaches and provides a more comprehensive explanation for the broad spectrum of health outcomes associated with breastfeeding. It also offers a biological rationale for investigating factors capable of perturbing this ecosystem, including maternal disease, environmental exposures, and pharmacological treatments administered during lactation. Breastfeeding is consistently associated with the establishment of gut microbial communities enriched in bifidobacteria and other taxa generally considered beneficial for infant health [7,11,36]. Such microbial configurations have been linked to enhanced intestinal resilience, improved epithelial maturation, and balanced immune development. While the mechanisms underlying these effects continue to be investigated, the available evidence supports the role of human milk as an important determinant of early microbial ecology [38]. Importantly, this biological ecosystem may be particularly relevant in vulnerable populations such as preterm infants. In these settings, breastfeeding has been associated with more favorable microbial colonization patterns and improved clinical outcomes compared with formula feeding [19,39,40]. The interaction between human milk components, microbial colonization, and immune maturation may therefore contribute to the resilience of the developing infant organism during periods of heightened biological vulnerability. Overall, current evidence supports the concept that human milk functions as a complex biological system rather than a simple nutritional substrate. The integration of prebiotic, microbial, immunological, and regulatory components appears to play a central role in shaping early-life physiology. Understanding this biological complexity is fundamental to evaluating how external influences, including antimicrobial therapies administered during lactation, may affect the maternal–infant ecosystem and potentially modify developmental trajectories (Figure 1).

### 2.2. The Microbiota–Gut–Brain Axis and Neurodevelopment

The microbiota–gut–brain (MGB) axis has emerged as a key conceptual framework for understanding the complex bidirectional communication occurring between the gastrointestinal microbiota and the central nervous system. This network involves neural, endocrine, metabolic, and immune pathways through which intestinal microorganisms may influence host physiology, behavior, and brain development [12,13]. Growing evidence suggests that early-life microbial colonization may contribute to neurodevelopmental processes, although the precise mechanisms and long-term clinical significance remain areas of active investigation. The first months of life represent a particularly critical period for both gut microbiome establishment and brain maturation. During this developmental window, microbial communities undergo rapid ecological succession, while the central nervous system experiences intense neuronal proliferation, synaptic formation, myelination, and functional organization [12,13]. The temporal overlap between these processes has led researchers to hypothesize that microbiome development may contribute to neurodevelopment through a variety of biological pathways. Experimental studies have demonstrated that gut microorganisms can influence host physiology through the production of metabolites, including short-chain fatty acids, neurotransmitter precursors, and other bioactive compounds capable of modulating immune and neuroendocrine signaling [12]. In addition, microbial communities interact extensively with the host immune system, contributing to the regulation of inflammatory pathways that may influence neural development and function. These findings have provided biologically plausible mechanisms supporting the role of the microbiota as an important component of early-life developmental programming. Within this context, human milk may act as an important modulator of the MGB axis. Through the combined effects of HMOs, immunological factors, microbial communities, and regulatory molecules, breastfeeding contributes to the establishment of a microbial environment that differs substantially from that observed in formula-fed infants [5,6,7,8,9,10,11]. Breastfed infants typically exhibit a microbiota enriched in Bifidobacterium species and other microorganisms capable of metabolizing HMOs and producing metabolites involved in host–microbe communication [6,11,36]. These observations suggest a potential indirect pathway through which human milk may influence neurodevelopmental processes. Interest in the relationship between breastfeeding and neurodevelopment extends beyond mechanistic considerations. Numerous epidemiological studies have reported associations between breastfeeding and improved outcomes in measures of cognitive performance, educational achievement, language development, and behavioral functioning [16,17,18,19,41,42]. A frequently cited systematic review and meta-analysis by Horta and colleagues reported a modest but statistically significant association between breastfeeding and higher intelligence quotient (IQ) scores later in life. Specifically, Horta and colleagues quantified this effect, demonstrating that breastfed subjects achieved a pooled mean advantage of 3.44 IQ points (95% CI: 2.30 to 4.58) compared to non-breastfed controls [16]. Similarly, studies conducted in preterm populations have suggested that exposure to human milk may be associated with favorable neurodevelopmental outcomes, including improvements in cognitive, language, and motor development [19]. However, interpretation of these findings requires substantial caution. Most of the available evidence derives from observational studies, which are inherently susceptible to confounding. Maternal intelligence, educational attainment, socioeconomic status, parenting practices, household environment, and healthcare access may independently influence both breastfeeding practices and developmental outcomes [17,18,41]. Although many investigations attempt to adjust for these factors, complete elimination of residual confounding remains difficult. Consequently, current evidence does not support the conclusion that breastfeeding directly and universally causes improved neurodevelopmental outcomes. Rather, the available literature supports a consistent association that is biologically plausible and likely mediated through multiple interacting mechanisms, including optimal nutrition, immune protection, maternal–infant interaction, epigenetic regulation, and microbiome-related effects [14,15,17]. Recognition of these limitations is particularly important when discussing the potential consequences of microbiome perturbations. Recent advances in epigenetics have added another dimension to the understanding of the relationship between human milk and neurodevelopment. Bioactive molecules contained within human milk, including microRNAs, extracellular vesicles, and other regulatory factors, may influence gene expression and developmental pathways involved in neural function [15,37,43]. Although these findings remain preliminary, they support the broader concept that human milk acts not simply as a nutrient source but as a biologically active signaling system capable of contributing to developmental regulation. The growing interest in the MGB axis has also generated concerns regarding factors capable of disrupting normal microbiome assembly during early life. Antibiotic exposure represents one of the most frequently discussed examples. Several studies have demonstrated that maternal or neonatal antibiotic administration may alter the composition of the infant microbiota, often reducing beneficial bacterial populations and modifying microbial diversity [20,21,22,23,24]. Such observations have raised the possibility that antibiotic-associated dysbiosis could influence gut–brain communication pathways. Nevertheless, the degree to which these microbiological alterations translate into clinically meaningful neurodevelopmental consequences remains uncertain. Most available studies evaluate microbiological endpoints rather than long-term functional outcomes. Furthermore, the observed changes often vary according to antibiotic class, treatment duration, timing of exposure, infant characteristics, and breastfeeding practices. Direct evidence linking microbiome alterations resulting from maternal antibiotic therapy during lactation to measurable neurodevelopmental impairment in humans remains limited. Current evidence therefore supports a balanced interpretation. The MGB axis provides a biologically credible framework through which human milk, microbiome development, and host physiology may interact during infancy. However, many proposed mechanisms remain incompletely understood, and substantial gaps persist between mechanistic observations and demonstrated clinical outcomes. Future longitudinal investigations incorporating microbiome profiling, metabolomics, neuroimaging, and standardized neurodevelopmental assessments will be essential to clarify these relationships. Taken together, the available literature supports the view that maintenance of a healthy maternal–infant microbial ecosystem may represent an additional consideration when evaluating pharmacological interventions during lactation. However, concerns regarding microbiome perturbation should not be interpreted as evidence that clinically indicated maternal antibiotic therapy causes adverse neurodevelopmental outcomes. Rather, they reinforce the need for evidence-based antimicrobial stewardship strategies aimed at optimizing therapy while minimizing unnecessary exposure. Understanding the biological interactions occurring along the microbiota–gut–brain axis provides an important conceptual foundation for clinical decision-making during lactation. These observations support further investigation into microbiome-conscious prescribing without overstating the current level of evidence regarding causality. Such an approach is consistent with both antimicrobial stewardship principles and the broader objective of preserving breastfeeding whenever clinically feasible.

### 2.3. Clinical Management, Antimicrobial Stewardship, and Lactation Pharmacotherapy

Anti-infective agents are among the most frequently prescribed medications during pregnancy, the postpartum period, and early infancy. Maternal infections such as mastitis, endometritis, urinary tract infections, surgical site infections, and other bacterial diseases often require prompt antimicrobial treatment to prevent severe maternal complications and ensure favorable clinical outcomes [20,21,22,23,24,25]. Therefore, the management of anti-infective therapies during lactation requires a careful balance between effective infection control and preservation of breastfeeding whenever clinically feasible. Historically, the safety evaluation of medications during breastfeeding has focused primarily on toxicological considerations, including drug transfer into human milk and the risk of direct adverse events in exposed infants [23,24,25,44,45,46]. Most antibiotics commonly prescribed during lactation are considered compatible with breastfeeding, and current recommendations generally discourage unnecessary interruption of breastfeeding when maternal treatment is required [21,23,44,47,48]. In recent years, however, growing attention has been directed toward the ecological consequences of antimicrobial exposure on the developing maternal–infant microbiome. The emerging concept of microbiome-conscious prescribing extends traditional pharmacological safety considerations by recognizing that medications may exert biological effects beyond direct toxicity. Antibiotics may alter microbial communities through several mechanisms, including direct infant exposure, modification of maternal microbiota, alterations in milk-associated microbial populations, and indirect effects on microbial colonization processes [20,21,22,23,24]. Several studies have reported reductions in microbial diversity and changes in the abundance of key bacterial taxa following maternal or neonatal antibiotic exposure [20,21,22,23,24]. In particular, decreases in Bifidobacterium populations have been observed after exposure to certain broad-spectrum antibiotic regimens [21,22]. Since bifidobacteria are considered important components of the microbial ecosystem typical of breastfed infants, these observations have generated concern regarding the potential consequences of antibiotic-associated dysbiosis. Nevertheless, caution is required when interpreting these findings. Most available studies focus on microbiological endpoints rather than clinically meaningful outcomes. Furthermore, substantial heterogeneity exists regarding study populations, antibiotic classes, treatment duration, microbiome assessment methods, and follow-up periods. Consequently, although microbiome alterations following antibiotic exposure appear biologically plausible and reasonably well documented, their long-term clinical significance remains incompletely defined [22,23,24]. From a practical clinical perspective, the available evidence strongly supports the principle that clinically indicated antimicrobial therapy should not be withheld because of theoretical concerns regarding microbiome disruption. Untreated maternal bacterial infections may result in significant morbidity and, in some cases, severe complications affecting both mother and infant. Therefore, effective infection management remains the primary therapeutic objective [21,25]. Within this framework, antimicrobial stewardship (AMS) offers an evidence-based strategy to optimize prescribing decisions. Initially developed to address antimicrobial resistance, AMS has evolved into a broader approach aimed at maximizing therapeutic effectiveness while minimizing avoidable adverse effects and inappropriate antimicrobial exposure [20,26,27,28,29,49,50]. In the context of lactation, four stewardship principles deserve particular attention (Figure 2). When applying these principles, clinicians must carefully distinguish established stewardship practices from emerging microbiome-oriented hypotheses. Diagnostic appropriateness, optimization of dosage, and utilization of the shortest clinically effective duration are evidence-based, established pillars of AMS. Conversely, ‘microbiome-conscious prescribing’—the selection of specific agents solely to preserve microbial diversity—remains a theoretical and emerging research area. While mechanistically plausible, current clinical evidence is insufficient to recommend altering first-line, guideline-concordant antibiotic therapies strictly for microbiome preservation.

#### Antibiotic-Specific Clinical Perspectives During Lactation

The impact of maternal antibiotics on the infant microbiome heavily depends on the specific pharmacokinetic properties of the drug class.

- Penicillins and Cephalosporins: These beta-lactams generally exhibit very low transfer into human milk (Relative Infant Dose [RID] typically <1–2%) and are degraded by the infant’s gastric acid, resulting in poor oral bioavailability. While toxicologically safe, they can cause transient alterations in the infant gut flora, occasionally manifesting as mild infant diarrhea.
- Macrolides: Drugs like erythromycin and azithromycin have slightly higher lipid solubility and milk-to-plasma ratios, leading to higher RIDs (up to 2–3%). Their broader spectrum can exert more pronounced selective pressure on the infant’s Bifidobacterium populations compared to narrow-spectrum penicillins.
- Tetracyclines: Although traditionally avoided due to concerns regarding infant dental staining, short courses of doxycycline are now considered acceptable. Tetracyclines strongly bind to calcium in human milk, which drastically reduces their oral bioavailability in the infant gut, thereby potentially limiting direct microbiological disruption in the infant compared to unbound drugs.

### 2.4. Diagnostic Appropriateness

Antibiotics should be prescribed only when there is reasonable clinical or microbiological evidence supporting a bacterial infection. Avoiding antibiotic use in viral infections and self-limiting conditions remains one of the most effective antimicrobial stewardship interventions and contributes directly to reducing antimicrobial resistance and unnecessary microbiome disturbance [20,26,48,51].

### 2.5. Selection of the Most Appropriate Agent

When antimicrobial treatment is required, clinicians should preferentially select agents with established safety profiles during lactation. Whenever clinically appropriate, narrow-spectrum antibiotics should be favored over broader-spectrum alternatives in order to reduce collateral ecological effects on commensal microbial communities [21,23,24]. This recommendation aligns with both antimicrobial stewardship principles and the broader objective of preserving microbial diversity while maintaining therapeutic efficacy.

### 2.6. Optimization of Dosage and Duration

Appropriate dosage selection and limitation of treatment duration to the shortest clinically effective regimen are fundamental components of stewardship programs [48,49]. Reducing unnecessary antimicrobial exposure may help minimize selective pressure on both pathogenic and commensal microorganisms while maintaining successful infection control. Although direct evidence specifically evaluating breastfeeding-associated microbiome outcomes remains limited, treatment optimization represents a low-risk intervention supported by substantial stewardship literature.

### 2.7. Multidisciplinary Management

Optimal prescribing decisions during lactation frequently require collaboration among obstetricians, pediatricians, infectious disease specialists, lactation consultants, and clinical pharmacists [21,44,47,48]. Multidisciplinary evaluation facilitates individualized risk-benefit assessment and may reduce unnecessary recommendations to discontinue breastfeeding. Such collaboration is particularly important in complex clinical situations involving preterm infants, neonatal intensive care unit patients, recurrent maternal infections, or prolonged antimicrobial therapy.

Patient counseling represents another essential component of clinical management. Mothers receiving antibiotic therapy often express concern regarding potential adverse effects on their infants, which may lead to premature interruption of breastfeeding. Healthcare professionals should therefore provide clear, balanced, and evidence-based information regarding medication safety during lactation [21,23,44,45]. Current evidence supports a reassuring clinical message: most commonly used antibiotics are compatible with breastfeeding, and interruption of breastfeeding is rarely necessary [21,44,47,48]. Moreover, breastfeeding itself may contribute to microbiome resilience through the continued provision of HMOs, immunological factors, and beneficial microbial exposures [5,6,7,8,22]. Accordingly, counseling should emphasize both the importance of completing clinically indicated treatment and the benefits of maintaining breastfeeding whenever possible. An emerging area of investigation concerns interventions designed to mitigate antibiotic-associated dysbiosis. Several studies have explored the potential role of probiotics, prebiotics, and nutritional strategies in supporting microbiome recovery following antimicrobial exposure [52,53]. Although preliminary results are encouraging, significant heterogeneity exists regarding probiotic strains, treatment protocols, study populations, and clinical endpoints. At present, the available evidence remains insufficient to recommend routine probiotic supplementation for all breastfeeding mothers receiving antibiotics [52,53]. Additional well-designed randomized controlled trials are needed before definitive clinical recommendations can be formulated. To facilitate interpretation of the available literature, major recommendations can be categorized according to the current strength of evidence (Table 1).

Taken together, current evidence supports a balanced and pragmatic clinical approach. Maternal infections should be diagnosed and treated appropriately, breastfeeding should generally be preserved, and antimicrobial stewardship principles should be integrated into routine clinical decision-making. Although microbiome disruption represents an emerging area of scientific interest, current data do not justify routine discontinuation of breastfeeding during antibiotic therapy. Rather, they reinforce the value of evidence-based prescribing practices aimed at minimizing unnecessary antimicrobial exposure while maintaining effective maternal care. This stewardship-oriented framework aligns with the broader One Health perspective, which recognizes the interconnected nature of human health, microbial ecology, and antimicrobial resistance. By integrating lactation pharmacology, microbiome science, and antimicrobial stewardship, clinicians can optimize therapeutic outcomes for both mothers and infants while preserving the biological integrity of the maternal–infant ecosystem.

## 3. Discussion and Future Directions

The present review highlights the evolving understanding of human milk as a complex biological system that contributes to infant health through the interaction of nutritional, immunological, microbial, and regulatory components. Rather than acting as an isolated source of nutrients, human milk appears to function as an integrated biological matrix capable of influencing host physiology during a critical developmental period [3,4,30]. This systems-based perspective is increasingly supported by studies demonstrating coordinated interactions among human milk oligosaccharides, immune mediators, microorganisms, extracellular vesicles, and other bioactive compounds [3,4,5,6,7,8]. One of the most consistent findings across the literature is the association between breastfeeding and the development of a gut microbiota enriched in bifidobacteria and other microorganisms typically regarded as beneficial for infant health [7,8,9,10,11,34]. Such microbial patterns have been associated with improved intestinal barrier function, balanced immune maturation, and resistance against pathogenic colonization. These observations provide biological plausibility for many of the short-term health benefits attributed to breastfeeding and support the concept that modulation of microbial colonization represents an important mechanism through which human milk contributes to infant development. The growing interest in the microbiota–gut–brain axis has further expanded this framework by suggesting potential links between microbiome development and neurodevelopmental outcomes [12,13]. Experimental evidence indicates that intestinal microorganisms may influence neural and behavioral processes through immune regulation, microbial metabolite production, endocrine signaling, and interactions with the central nervous system. These findings have generated substantial interest in understanding whether breastfeeding-related alterations in the microbiome could contribute to cognitive and neurodevelopmental outcomes observed later in life. However, one of the principal challenges within this field concerns the distinction between biological plausibility and demonstrated clinical causality. Although numerous observational studies report favorable cognitive, behavioral, and educational outcomes among breastfed children, most available evidence remains susceptible to confounding [16,17,18,41,42]. Maternal education, intelligence, socioeconomic status, parenting practices, family environment, and healthcare access may independently influence both breastfeeding behavior and developmental outcomes. Consequently, while breastfeeding is consistently associated with improved neurodevelopmental measures, the magnitude and relative contribution of individual mechanisms remain difficult to quantify. This limitation is particularly relevant when considering the hypothesized role of the microbiome as an intermediary factor. Most studies linking microbiome composition to neurodevelopment rely on mechanistic models, preclinical experiments, or observational human data. Although these investigations provide valuable insights, they do not allow definitive conclusions regarding causality. Therefore, statements suggesting that specific microbiome alterations directly cause neurodevelopmental consequences should be interpreted cautiously and within the context of the current limitations of the available evidence. A second major challenge concerns the study of human milk microbiota itself. Despite substantial advances in sequencing technologies, considerable methodological heterogeneity remains across investigations [9,10,31,32]. Differences in sample collection methods, storage conditions, DNA extraction protocols, sequencing platforms, analytical pipelines, and contamination-control procedures frequently limit direct comparisons between studies. As a result, important questions remain regarding the composition, origin, stability, and biological significance of milk-associated microbial communities. Similarly, evidence regarding the effects of antimicrobial exposure during lactation must be interpreted carefully. Several studies have demonstrated that maternal or neonatal antibiotic treatment may alter microbial composition and reduce the abundance of bacterial taxa commonly associated with breastfeeding [20,21,22,23,24]. These findings support concerns regarding the ecological consequences of antimicrobial therapy during critical periods of microbiome development. Nevertheless, the long-term clinical implications of these microbiome alterations remain largely uncertain. Importantly, most studies evaluating antibiotic-associated dysbiosis focus on microbiological outcomes rather than clinically relevant endpoints. Changes in microbial diversity or abundance do not necessarily translate into measurable health consequences, and the resilience of infant microbial ecosystems following antibiotic exposure remains incompletely understood. Furthermore, observed effects vary according to antibiotic class, dosing regimen, duration of treatment, route of administration, infant age, feeding practices, and host-related factors. These sources of heterogeneity complicate attempts to generate universal clinical conclusions. Despite these limitations, several clinically meaningful observations emerge from the available evidence. First, there is currently no scientific basis for routinely recommending cessation of breastfeeding during most maternal antibiotic treatments [21,23,44,47,48]. Second, the potential ecological impact of antibiotics provides additional support for applying antimicrobial stewardship principles during lactation. Third, the preservation of breastfeeding should remain a central objective whenever clinically appropriate, given its well-established benefits for infant health and development. The concept of microbiome-conscious prescribing represents an important evolution of antimicrobial stewardship. Traditionally, stewardship programs have focused on optimizing therapeutic efficacy while reducing antimicrobial resistance. The emerging literature suggests that stewardship initiatives may also contribute to preserving the developing maternal–infant microbial ecosystem by promoting targeted therapy, minimizing unnecessary exposure, and encouraging evidence-based prescribing practices [26,27,28,29,48,49]. Although further evidence is required to define the clinical relevance of these ecological effects, the approach is consistent with the principles of prudent antimicrobial use. Another important consideration is communication with patients and families. Public awareness regarding microbiome health has increased substantially in recent years, occasionally leading to exaggerated concerns regarding the potential harms of antibiotic exposure. Healthcare professionals should therefore provide balanced counseling that acknowledges current scientific uncertainties while emphasizing that appropriate treatment of maternal infections remains essential. Failure to treat clinically significant infections may expose both mother and infant to substantially greater risks than the theoretical microbiome-related concerns currently described in the literature. From a broader public health perspective, the integration of antimicrobial stewardship, breastfeeding promotion, and microbiome science aligns closely with the principles of the One Health framework [26,28,50,51]. This framework emphasizes the interconnected relationship between human health, microbial ecology, and antimicrobial resistance. In this context, stewardship should not be viewed solely as a strategy to combat resistance but also as a means of supporting responsible pharmacological interventions during sensitive developmental periods. Overall, the available evidence supports a balanced interpretation of the relationships among breastfeeding, microbiome development, antimicrobial exposure, and neurodevelopment. Human milk clearly contributes to shaping the early-life microbial environment, and antimicrobial exposure may influence this process. However, important uncertainties remain regarding the magnitude, persistence, and clinical consequences of these effects. Future investigations should therefore focus on generating robust longitudinal data capable of linking microbiological observations with meaningful clinical outcomes. The current state of knowledge supports a pragmatic clinical message: maternal infections should be treated appropriately, breastfeeding should be maintained whenever possible, and antimicrobial stewardship principles should be systematically applied. Such an approach allows clinicians to optimize maternal care while preserving the many established benefits of breastfeeding and avoiding unsupported assumptions regarding causality.

Despite significant advances in the understanding of human milk biology, infant microbiome development, and the microbiota–gut–brain axis, important scientific uncertainties remain. These limitations should be recognized when interpreting current evidence and developing future clinical recommendations. One of the principal knowledge gaps concerns the characterization of the human milk microbiome itself. Although numerous studies have demonstrated the presence of microbial communities in human milk, substantial variability exists among reports regarding microbial composition, diversity, and abundance [9,10,31,32]. This variability is influenced by differences in maternal characteristics, geographical location, mode of delivery, lactation stage, feeding practices, and methodological approaches. Furthermore, technical issues related to sample collection and contamination control continue to complicate interpretation of findings. As a result, a universally accepted definition of the physiological human milk microbiome has yet to be established. Another major challenge involves understanding the dynamics of microbial transfer between mother and infant. While evidence suggests that breastfeeding contributes to infant gut colonization, the relative contribution of milk-derived microorganisms compared with environmental exposures, maternal skin microbiota, and oral microbial exchange remains poorly quantified [7,8,9,10]. Future studies employing standardized methodologies and integrated longitudinal sampling strategies will be essential to clarify these interactions. A second important area of uncertainty concerns the long-term consequences of microbiome alterations during infancy. Current evidence indicates that maternal and neonatal antibiotic exposure can modify microbial composition and diversity [20,21,22,23,24]. However, the persistence of these changes and their relationship with future clinical outcomes remain incompletely understood. Most available studies evaluate short-term microbiological endpoints rather than long-term health consequences. Consequently, whether transient microbial perturbations translate into measurable effects on immune, metabolic, or neurological development remains uncertain. Similarly, considerable uncertainty surrounds the relationship between microbiome development and neurodevelopmental outcomes. Experimental and translational research has identified multiple biologically plausible mechanisms linking gut microorganisms to brain development [12,13]. However, direct evidence from human populations remains limited. Most available studies evaluate associations rather than causation and are subject to substantial confounding. The challenge of disentangling microbiome effects from nutritional, immunological, genetic, environmental, and socioeconomic influences remains one of the most important methodological obstacles in this field. Future research should therefore prioritize large prospective longitudinal cohorts integrating microbiome profiling with comprehensive developmental assessment. Such studies should simultaneously evaluate nutritional exposures, maternal health, environmental factors, microbial composition, metabolomic markers, immune responses, and neurodevelopmental outcomes. Multidimensional approaches of this type may help clarify causal pathways and improve understanding of the biological mechanisms involved. Another important research priority concerns antimicrobial stewardship during lactation. Although current stewardship recommendations are largely extrapolated from broader antimicrobial resistance frameworks, evidence specifically addressing stewardship interventions in breastfeeding populations remains limited [26,48,49,51]. Future studies should investigate whether microbiome-conscious prescribing practices can meaningfully influence infant microbial development while maintaining equivalent clinical effectiveness. Additional research is also required regarding differences among specific antibiotic classes. Existing studies frequently evaluate antibiotic exposure as a single category despite substantial variation in antimicrobial spectrum, pharmacokinetics, transfer into human milk, and ecological impact on microbial communities [21,22,23,24,25]. Comparative investigations examining individual antibiotic classes may help identify prescribing strategies capable of minimizing microbiome disruption without compromising therapeutic efficacy. The potential role of microbiome-supportive interventions also warrants further investigation. Probiotics, prebiotics, synbiotics, and nutritional strategies have shown promise in preliminary studies; however, current evidence remains heterogeneous and insufficient to support routine implementation [52,53]. Future randomized controlled trials should focus on identifying effective interventions, optimal timing, appropriate target populations, and clinically meaningful outcomes. Another emerging field involves the study of extracellular vesicles, non-coding RNAs, and epigenetic mechanisms present in human milk [15,37,43]. These bioactive components may act as signaling molecules capable of influencing immune programming and neurodevelopment. Nevertheless, current evidence remains largely mechanistic, and their clinical significance has not yet been fully established. Advances in systems biology, transcriptomics, and multi-omics technologies may facilitate further progress in this area. Importantly, future investigations should strive for greater methodological standardization. The implementation of uniform protocols for sample collection, microbiome analysis, outcome definition, and reporting standards would substantially improve comparability among studies and strengthen evidence synthesis efforts. Such standardization is particularly important given the rapidly expanding body of literature in this field. From a clinical perspective, future evidence should focus not only on biological plausibility but also on measurable patient-centered outcomes. Demonstrating microbiological changes is important; however, establishing whether these changes translate into meaningful differences in infant health, neurodevelopment, immune function, or disease risk is ultimately more relevant for clinical decision-making. Overall, the field is transitioning from descriptive microbiome research toward a more integrated understanding of human milk as a biologically active system influencing multiple developmental pathways. Continued progress will require interdisciplinary collaboration among neonatologists, pediatricians, microbiologists, pharmacologists, nutrition scientists, epidemiologists, and public health specialists. Such collaborative efforts may ultimately support the development of evidence-based guidelines that integrate breastfeeding promotion, antimicrobial stewardship, and microbiome preservation within a unified framework of early-life healthcare.

## 4. Conclusions

Human milk is an integrated biological matrix that shapes early microbial colonization and supports infant immune maturation. While the microbiota–gut–brain axis provides a biologically plausible mechanism for how early-life microbial development may influence neurodevelopment, this review underscores that direct clinical evidence linking maternal antibiotic therapy during lactation to adverse infant neurodevelopmental outcomes remains severely limited. Currently, microbiome preservation strategies have not been proven to prevent neurodevelopmental impairment.

Maternal anti-infective therapy during lactation is a clinical necessity that must be guided by established Antimicrobial Stewardship (AMS) principles [54]. Optimization of prescribing practices—through accurate diagnosis and the shortest effective treatment duration—ensures effective maternal infection control while minimizing unnecessary antibiotic exposure. Although emerging concepts such as microbiome-conscious prescribing are mechanistically appealing, they should not supersede established infectious disease guidelines. Ultimately, the continuity of breastfeeding should be preserved during most maternal antibiotic treatments, as human milk continues to provide indispensable resilience to the infant ecosystem.

## Figures and Tables

**Figure 1:**
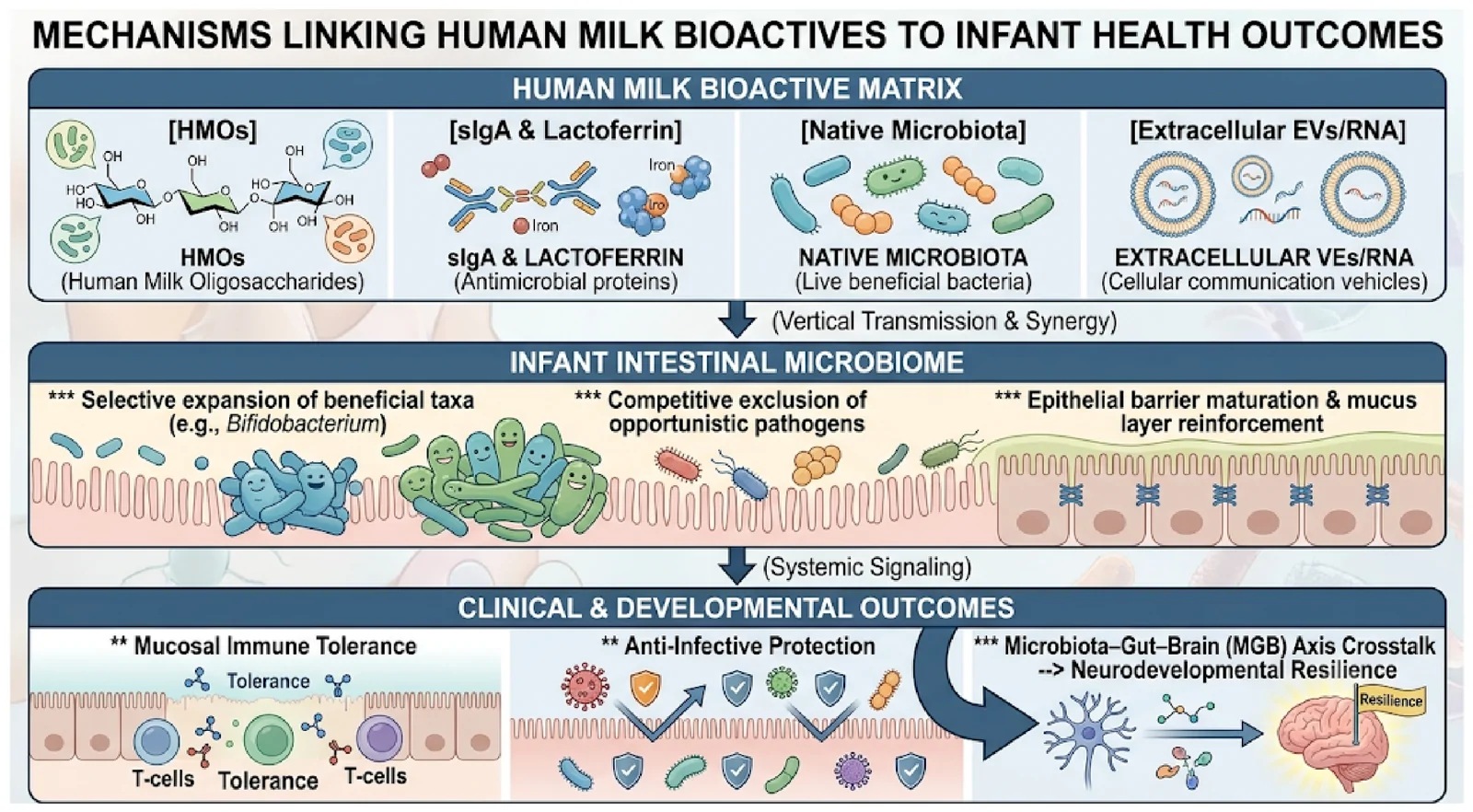
Human milk as an integrated biological system and modulator of early intestinal resilience. Human milk delivers an extensive repertoire of bioactive components—including human milk oligosaccharides (HMOs), secretory immunoglobulin A (sIgA), antimicrobial proteins, extracellular vesicles, and a native microbiota—that synergistically direct infant gut colonization. Selective enrichment of beneficial taxa, notably Bifidobacterium, promotes epithelial barrier maturation, mucosal immune tolerance, and systemic signaling along the microbiota–gut–brain axis during the first 1000 days of life. Abbreviations: EVs, Extracellular Vesicles; HMOs, Human Milk Oligosaccharides; MGB, Microbiota–Gut–Brain; sIgA, Secretory Immunoglobulin A; ** clinical outcome *** infant microbiome. Note: The neurodevelopmental outcomes and the microbiota–gut–brain axis signaling depicted in this figure represent hypothesized mechanistic pathways based on preclinical and observational data, rather than established clinical endpoints.

**Figure 2:**
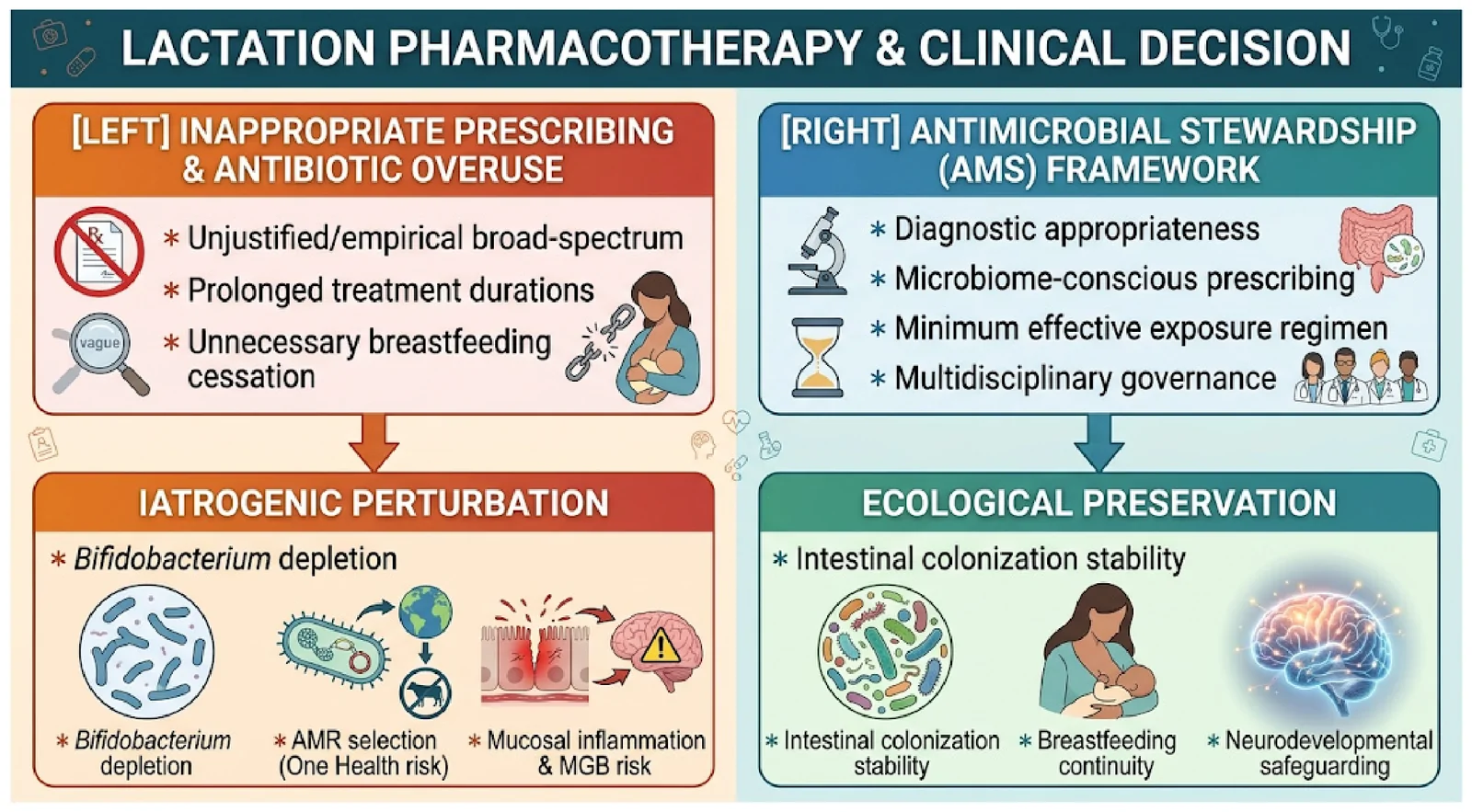
Antimicrobial Stewardship (AMS) and clinical management framework during lactation. Inappropriate antibiotic prescribing and empirical broad-spectrum overuse drive iatrogenic dysbiosis, deplete essential Bifidobacterium populations, foster antimicrobial resistance (AMR), and risk disrupting early microbiota–gut–brain signaling. (Right) An integrated AMS framework—grounded in diagnostic appropriateness, microbiome-conscious prescribing, minimum effective exposure, and multidisciplinary governance—preserves the maternal–infant microbial axis, ensures breastfeeding continuity, and aligns clinical pharmacotherapy with the World Health Organization’s One Health mandate. Abbreviations: AMR, Antimicrobial Resistance; AMS, Antimicrobial Stewardship; MGB, Microbiota–Gut–Brain; WHO, World Health Organization. Note: The neurodevelopmental outcomes and the microbiota–gut–brain axis signaling depicted in this figure represent hypothesized mechanistic pathways based on preclinical and observational data, rather than established clinical endpoints.

**Table 1:** Strength of evidence for major clinical recommendations during lactation. Evidence grading: High = supported by systematic reviews, clinical guidelines, and consistent observational evidence; Moderate = supported by observational and mechanistic evidence; Low = supported primarily by preliminary, heterogeneous, or indirect evidence.

Clinical Recommendation	Strength of Evidence
Continue breastfeeding during most maternal antibiotic treatments	High
Avoid unnecessary antibiotic prescriptions	High
Use microbiologically targeted therapy whenever feasible	Moderate
Prefer narrow-spectrum agents when clinically appropriate	Moderate
Consider microbiome effects during prescribing decisions	Moderate
Routine maternal probiotic supplementation to prevent dysbiosis	Low
Minimizing iatrogenic microbiome disruption to support physiological gut–brain signaling	Low

## Data Availability

All data and materials are available upon reasonable request.

## Abbreviations

AMR	Antimicrobial Resistance
AMS	Antimicrobial Stewardship
HMOs	Human Milk Oligosaccharides
IQ	Intelligence Quotient
MGB	Microbiota–Gut–Brain
miRNA	MicroRNA
ncRNA	Non-coding Ribonucleic Acid
NEC	Necrotizing Enterocolitis
PPIs	Proton Pump Inhibitors
sIgA	Secretory Immunoglobulin A
WHO	World Health Organization

## References

[1] Victora C.G., Bahl R., Barros A.J.D., Franca G.V.A., Horton S., Krasevec J., Murch S., Sankar M.J., Walker N., Rollins N.C. (2016). Breastfeeding in the 21st century: Epidemiology, mechanisms, and lifelong effect. Lancet.

[2] Ballard O., Morrow A.L. (2013). Human milk composition: Nutrients and bioactive factors. Pediatr. Clin. N. Am..

[3] Donovan S.M., Aghaeepour N., Andres A., Azad M.B., Becker M., Carlson S.E., Järvinen K.M., Lin W., Lönnerdal B., Slupsky C.M. (2023). Evidence for human milk as a biological system and recommendations for study design: A report from “Breastmilk Ecology: Genesis of Infant Nutrition (BEGIN)” Working Group 4. Am. J. Clin. Nutr..

[4] Smilowitz J.T., Allen L.H., Dallas D.C., McManaman J., Raiten D.J., Rozga M., Sela D.A., Seppo A., Williams J.E., Young B.E. (2023). Ecologies, synergies, and biological systems shaping human milk composition: A report from “Breastmilk Ecology: Genesis of Infant Nutrition (BEGIN)” Working Group 2. Am. J. Clin. Nutr..

[5] Carr L.E., Virmani M.D., Rosa F., Munblit D., Matazel K.S., Elolimy A.A., Yeruva L. (2021). Role of human milk bioactives on infants’ gut and immune health. Front. Immunol..

[6] Singh R.P., Niharika J., Kondepudi K.K., Bishnoi M., Tingirikari J.M.R. (2022). Recent understanding of human milk oligosaccharides in establishing infant gut microbiome and roles in immune system. Food Res. Int..

[7] Le Doare K., Holder B., Bassett A., Pannaraj P.S. (2018). Mother’s milk: A purposeful contribution to the development of the infant microbiota and immunity. Front. Immunol..

[8] Lyons K.E., Ryan C.A., Dempsey E.M., Ross R.P., Stanton C. (2020). Breast milk, a source of beneficial microbes and associated benefits for infant health. Nutrients.

[9] Notarbartolo V., Giuffrè M., Montante C., Corsello G., Carta M. (2022). Composition of human breast milk microbiota and its role in children’s health. Pediatr. Gastroenterol. Hepatol. Nutr..

[10] Edwards C.A., Van Loo-Bouwman C.A., Van Diepen J.A., Schoemaker M.H., Ozanne S.E., Venema K., Stanton C., Marinello V., Rueda R., Flourakis M. (2022). A systematic review of breast milk microbiota composition and evidence for transfer to the infant gut. Benef. Microbes.

[11] Walsh C., Lane J.A., van Sinderen D., Hickey R.M. (2020). Human milk oligosaccharides: Shaping the infant gut microbiota and supporting health. J. Funct. Foods.

[12] Wang Q., Yang Q., Liu X. (2023). The microbiota-gut-brain axis and neurodevelopmental disorders. Protein Cell.

[13] Frerichs N.M., de Meij T.G.J., Niemarkt H.J. (2024). Microbiome and its impact on fetal and neonatal brain development. Curr. Opin. Clin. Nutr. Metab. Care.

[14] de Weerth C., Aatsinki A.-K., Azad M.B., Bartol F.F., Bode L., Collado M.C., Dettmer A.M., Field C.J., Guilfoyle M., Hinde K. (2023). Human milk: From complex tailored nutrition to bioactive impact on child cognition and behavior. Crit. Rev. Food Sci. Nutr..

[15] Gialeli G., Panagopoulou O., Liosis G., Siahanidou T. (2023). Potential epigenetic effects of human milk on infants’ neurodevelopment. Nutrients.

[16] Horta B.L., Loret de Mola C., Victora C.G. (2015). Breastfeeding and intelligence: A systematic review and meta-analysis. Acta Paediatr..

[17] Walfisch A., Sermer C., Cressman A., Koren G. (2013). Breast milk and cognitive development: The role of confounders. BMJ Open.

[18] Bar S., Milanaik R., Adesman A. (2016). Long-term neurodevelopmental benefits of breastfeeding. Curr. Opin. Pediatr..

[19] Zhang R., Ying E., Wu X., Qin H., Guo Y., Guo X., Yu Z., Chen J. (2024). Breastfeeding and neurodevelopmental outcomes in preterm infants: A systematic review and meta-analysis. Front. Public Health.

[20] Zimmermann P., Curtis N. (2021). The impact of antibiotics on the infant gut microbiota. Curr. Opin. Infect. Dis..

[21] Reyman M., van Houten M.A., Watson R.L., Chu M.L.J.N., Arp K., de Waal W.J., Schiering I., Plötz F.B., Willems R.J.L., van Schaik W. (2022). Effects of early-life antibiotics on the developing infant gut microbiome and resistome: A randomized trial. Nat. Commun..

[22] Brockway M. (2024). The role of antibiotic exposure and the effects of breastmilk and human milk feeding on the developing infant gut microbiome. Front. Public Health.

[23] van Wattum J.J., Leferink T.M., Wilffert B., Ter Horst P.G.J. (2019). Antibiotics and lactation: An overview of relative infant doses and a systematic assessment of clinical studies. Basic Clin. Pharmacol. Toxicol..

[24] Thomas S.P., Denizer E., Zuffa S., Best B.M., Bode L., Chambers C.D., Dorrestein P.C., Liu G.Y., Momper J.D., Nizet V. (2023). Transfer of antibiotics and their metabolites in human milk: Implications for infant health and microbiota. Pharmacotherapy.

[25] Gong C., Bertagnolli L.N., Boulton D.W., Coppola P. (2024). A literature review of drug transport mechanisms during lactation. CPT Pharmacomet. Syst. Pharmacol..

[26] Velazquez-Meza M.E., Galarde-López M., Carrillo-Quiróz B., Alpuche-Aranda C.M. (2022). Antimicrobial resistance: One Health approach. Vet. World.

[27] Ferrara F., Zovi A., Nava E., Trama U., Vitiello A. (2023). SARS-CoV-2 caused a surge in antibiotic consumption causing a silent pandemic inside the pandemic. Ann. Pharm. Fr..

[28] Siachalinga L., Mufwambi W., Lee I.H. (2022). Impact of antimicrobial stewardship interventions to improve antibiotic prescribing for hospital inpatients in Africa: A systematic review and meta-analysis. J. Hosp. Infect..

[29] Baur D., Gladstone B.P., Burkert F., Carrara E., Foschi F., Döbele S., Tacconelli E. (2017). Effect of antibiotic stewardship on the incidence of antibiotic-resistant bacteria and Clostridioides difficile infection: A systematic review and meta-analysis. Lancet Infect. Dis..

[30] Christian P., Smith E.R., Lee S.E., Vargas A.J., Bremer A.A., Raiten D.J. (2021). The need to study human milk as a biological system. Am. J. Clin. Nutr..

[31] Lopez Leyva L., Brereton N.J.B., Koski K.G. (2020). Emerging frontiers in human milk microbiome research and suggested primers for 16S rRNA gene analysis. Comput Struct. Biotechnol. J..

[32] Selma-Royo M., Calvo-Lerma J., Bäuerl C., Esteban-Torres M., Cabrera-Rubio R., Collado M.C. (2022). Human milk microbiota: What did we learn in the last 20 years?. Microbiome Res. Rep..

[33] Moubareck C.A. (2021). Human milk microbiota and oligosaccharides: A glimpse into benefits, diversity, and correlations. Nutrients.

[34] Donovan S.M., Comstock S.S. (2016). Human milk oligosaccharides influence neonatal mucosal and systemic immunity. Ann. Nutr. Metab..

[35] Thai J.D., Gregory K.E. (2020). Bioactive factors in human breast milk attenuate intestinal inflammation during early life. Nutrients.

[36] Chichlowski M., De Lartigue G., German J.B., Raybould H.E., Mills D.A. (2012). Bifidobacteria isolated from infants and cultured on human milk oligosaccharides affect intestinal epithelial function. J. Pediatr. Gastroenterol. Nutr..

[37] Karlsson O., Rodosthenous R.S., Jara C., Brennan K.J., Wright R.O., Baccarelli A.A., Wright R.J. (2016). Detection of long non-coding RNAs in human breastmilk extracellular vesicles: Implications for early child development. Epigenetics.

[38] Davis E.C., Castagna V.P., Sela D.A., Hillard M.A., Lindberg S., Mantis N.J., Seppo A.E., Järvinen K.M. (2022). Gut microbiome and breast-feeding: Implications for early immune development. J. Allergy Clin. Immunol..

[39] Asbury M.R., Butcher J., Copeland J.K., Unger S., Bando N., Comelli E.M., Forte V., Kiss A., LeMay-Nedjelski L., Sherman P.M. (2020). Mothers of Preterm Infants Have Individualized Breast Milk Microbiota that Changes Temporally Based on Maternal Characteristics. Cell Host Microbe.

[40] Shama S., Asbury M.R., Kiss A., Bando N., Butcher J., Comelli E.M., Copeland J.K., Greco A., Kothari A., Sherman P.M. (2024). Mother’s milk microbiota is associated with the developing gut microbial consortia in very-low-birth-weight infants. Cell Rep. Med..

[41] Horwood L.J., Darlow B.A., Mogridge N. (2001). Breast milk feeding and cognitive development at 5 and 18 years. Arch. Dis. Child. Fetal Neonatal Ed..

[42] Strøm M., Mortensen E.L., Kesmodel U.S., Halldorsson T.I., Olsen J., Olsen S.F. (2019). Is breastfeeding associated with offspring IQ at age 5?. BMJ Open.

[43] Xu J., Shin J., McGee M., Unger S., Bando N., Sato J., Vandewouw M., Patel Y., Branson H.M., Paus T. (2022). Intake of mother’s milk and variation in DNA methylation of genes involved in neurodevelopment at 5.5 years of age. Am. J. Clin. Nutr..

[44] Hale T.W., Rowe H.E. (2023). Medications and Mothers’ Milk.

[45] National Library of Medicine (2025). Drugs and Lactation Database (LactMed^®^).

[46] Tezel Yalçın H., Yalçın N., Ceulemans M., Allegaert K. (2024). Drug safety during breastfeeding: A comparative analysis of FDA adverse event reports and LactMed^®^. Pharmaceuticals.

[47] Academy of Breastfeeding Medicine Clinical Protocols. Breastfeed Med. https://www.bfmed.org/protocols.

[48] Bar-Oz B., Bulkowstein M., Benyamini L., Greenberg R., Soriano I., Zimmerman D., Bortnik O., Berkovitch M. (2003). Use of antibiotic and analgesic drugs during lactation. Drug Saf..

[49] World Health Organization (2015). Global Action Plan on Antimicrobial Resistance.

[50] World Health Organization (2025). Antimicrobial Resistance.

[51] World Health Organization (2025). One Health Joint Plan of Action (2022–2026).

[52] Alemu B.K., Azeze G.G., Wu L., Lau S.L., Wang C.C., Wang Y. (2023). Effects of maternal probiotic supplementation on breast milk microbiome and infant gut microbiome and health: A systematic review and meta-analysis of randomized controlled trials. Am. J. Obstet. Gynecol. MFM.

[53] Parracho H., McCartney A.L., Gibson G.R. (2007). Probiotics and prebiotics in infant nutrition. Proc. Nutr. Soc..

[54] World Health Organization (2025). Infant and Young Child Feeding.

